# Mitochondrial regulator ATAD3A: a molecular determinant favoring head and neck cancer development

**DOI:** 10.18632/oncoscience.574

**Published:** 2023-04-04

**Authors:** Yong Teng

**Affiliations:** ^1^Department of Hematology and Medical Oncology, Winship Cancer Institute, Emory University, School of Medicine, Atlanta, GA 30322, USA; ^2^Wallace H. Coulter Department of Biomedical Engineering, Georgia Institute of Technology and Emory University, Atlanta, GA 30322, USA

**Keywords:** ATAD3A, ERK1/2, cancer, mitochondria, anti-cancer target

## INTRODUCTION

In addition to their role in energy metabolism, mitochondria play important roles in other cellular processes, such as apoptosis, calcium signaling, and the synthesis of certain biomolecules. Mitochondria have also been implicated in the development and progression of cancer [1, 2]. In some cases, cancer cells may overproduce certain mitochondrial proteins, known as oncoproteins, that contribute to the uncontrolled growth and survival of cancer cells. Targeting these oncoproteins could offer a novel approach to developing effective cancer therapeutics. However, non-specific targeting of mitochondrial functions has significant unwarranted effects on normal cell growth, and it could lead to unwanted side effects. Therefore, it is important to develop refined strategies that can specifically target oncoproteins that are physically localized to mitochondria in cancer cells.

The mitochondrial ATPase family AAA domain-containing protein 3 (ATAD3) belongs to the AAA+ superfamily of ATPases and is involved in various cellular processes [3–5]. There are three family members of ATAD3, with ATAD3A being the ancestral form and ATAD3B and ATAD3C being duplicates of ATAD3A. Structurally, ATAD3A spans the mitochondrial outer and inner membranes and regulates dynamic interactions between the two membranes, which are sensed by the mitochondrial fission machinery. ATAD3A is involved in regulating a wide range of physiological and pathological responses, including mitochondrial dynamics, nucleoid organization, signal transduction, and cholesterol metabolism [3–5]. Mutations in ATAD3A can cause mitochondrial disease, and elevated ATAD3A expression in various cancer types is associated with poor patient outcomes [3–6]. Our previous study has demonstrated that ATAD3A overexpression in breast cancer cells promoted metastasis to the lung and liver in a mouse model, while its knockdown suppressed metastasis [4]. ATAD3A has also been linked to epithelial-mesenchymal transition (EMT), a process by which cancer cells lose their epithelial characteristics and acquire mesenchymal properties, enabling them to invade and migrate. The exact mechanisms by which ATAD3A promotes cancer metastasis are not fully understood, but it is thought to be related to its regulation of mitochondrial metabolism and dynamics, as well as its interaction with WASF3-dependent metastatic cascade involved in cancer progression [4].

Head and neck squamous cell carcinoma (HNSCC) represents a serious and potentially life-threatening disease if not diagnosed and treated promptly. The five-year survival rate for HNSCC is around 60%, but this can vary widely depending on the specific type of cancer and other individual factors [7, 8]. Our recently published study has shown that ATAD3A is highly expressed in HNSCC tissues and cell lines [9]. Loss of ATAD3A expression suppresses HNSCC cell growth and induces tumor regression in orthotopic tumor-bearing mice, whereas gain of ATAD3A expression has the opposite effect. Mechanistically, the tumor suppression induced by overexpression of the Walker A dead mutant of ATAD3A (K358) produces a potent dominant-negative effect due to defective ATP binding. Furthermore, ATAD3A binds to ERK1/2 in the mitochondria of HNSCC cells in the presence of VDAC1, a protein primarily located in the outer mitochondrial membrane. The ATAD3A-VDAC1 interaction is essential for the activation of mitochondrial ERK1/2 signaling. Most importantly, the ATAD3A-ERK1/2 signaling axis drives HNSCC development in a RAS-independent manner, and thus tumor suppression is more effective when ATAD3A knockout is combined with RAS inhibitor treatment.

Coupled with rigorous *in vitro* and *in vivo* validations, this study gains unprecedented insights into the mitochondrial oncogenic signaling mediated by ATAD3A as well as unraveling it as a promising molecular target for anti-HNSCC [9]. Blockade of the ERK1/2 pathway is an attractive approach for the treatment of malignancies with increased ERK1/2 activity [10, 11]. Since ATAD3A-mediated ERK1/2 phosphorylation is RAS-independent, it will be important to harness this signaling pathway along with the cytoplasmic ERK1/2 cascade to develop more effective anti-cancer therapies. Salirasib is an orally available, targeted RAS inhibitor being developed for treatment of a wide range of malignancies. However, clinical trials of salirasib have shown mixed results, with some studies suggesting that the drug may be effective in some patients, while others have not shown significant benefits [12, 13]. Our animal data have shown the therapeutic promise of this combination by adding salirasib treatment to the setting of ATAD3A gene suppression [9]. Given that there are currently no drugs available to inhibit ATAD3A, our study highlights the need to expand the development of anti-HNSCC therapies to include novel anti-ATAD3A strategies. The promising nature of the results of this study also suggests that targeting the WA motif of ATAD3A or the ATAD3A-ERK1/2 signaling node may attenuate the oncogenic function of ATAD3A in cancer cells [9]. Follow-up studies in our laboratory include the design of highly specific stapled peptides [14–16] to incorporate a hydrophobic staple at the α-helical interface between ATAD3A and ERK1/2. This novel approach can specifically disrupt the ATAD3A-ERK1/2 interaction and subsequently block mitochondrial ATAD3A-ERK1/2 oncogenic signaling.

Although there are many molecules and pathways (including PKC, WASF3, and FAT1) that regulate ATAD3A expression and function at different levels, further research is needed to fully understand the complex signaling networks of ATAD3A. This includes identifying upstream regulators and downstream effectors of ATAD3A, as well as the molecular mechanisms underlying its interactions with other mitochondrial proteins and effectors spatially and temporally. Nevertheless, developing targeted therapies that specifically inhibit ATAD3A in cancer cells while sparing normal cells will be a challenging but critical task. This will require a detailed understanding of the biological and pathological roles of ATAD3A. Continued research on ATAD3A and its regulation will provide valuable insights into the molecular mechanisms underlying cancer progression and the development of effective anti-cancer therapeutics.
